# Artificial Intelligence-Based Diagnosis of Diabetes Mellitus: Combining Fundus Photography with Traditional Chinese Medicine Diagnostic Methodology

**DOI:** 10.1155/2021/5556057

**Published:** 2021-04-20

**Authors:** Yang Xiang, Lai Shujin, Chang Hongfang, Wen Yinping, Yu Dawei, Dong Zhou, Li Zhiqing

**Affiliations:** ^1^Beijing University of Chinese Medicine, 100000, China; ^2^EVision Technology (Beijing) Co. LTD, 100000, China; ^3^Department of Traditional Chinese Medicine, Tuha Petroleum Hospital, 839009, China; ^4^Tianjin Medical University, 300384, China; ^5^Tianjin Aier Eye Hospital, 300190, China; ^6^Tianjin Jinghai District Hospital, 301600, China; ^7^Tianjin Key Laboratory of Retinal Functions and Diseases, Tianjin Branch of National Clinical Research Center for Ocular Disease, Eye Institute and School of Optometry, Tianjin Medical University Eye Hospital, 300384, China

## Abstract

In this study, we propose a technique for diagnosing both type 1 and type 2 diabetes in a quick, noninvasive way by using equipment that is easy to transport. Diabetes mellitus is a chronic disease that affects public health globally. Although diabetes mellitus can be accurately diagnosed using conventional methods, these methods require the collection of data in a clinical setting and are unlikely to be feasible in areas with few medical resources. This technique combines an analysis of fundus photography of the physical and physiological features of the patient, namely, the tongue and the pulse, which are used in Traditional Chinese Medicine. A random forest algorithm was used to analyze the data, and the accuracy, precision, recall, and F1 scores for the correct classification of diabetes were 0.85, 0.89, 0.67, and 0.76, respectively. The proposed technique for diabetes diagnosis offers a new approach to the diagnosis of diabetes, in that it may be convenient in regions that lack medical resources, where the early detection of diabetes is difficult to achieve.

## 1. Introduction

Diabetes mellitus is a significant chronic disease that affects 463 million people globally and 116.4 million people in China as of 2019 [1]. This disease is mainly classified into two groups: type 1 diabetes and type 2 diabetes. According to reference [2], the cost of diabetes is still a burden, even for patients in a relatively wealthy city in China.

There is a correlation between eye conditions and diabetes. For example, diabetic retinopathy is the most common critical complication of diabetes. Moreover, during the first two decades of disease progression, nearly all patients with type 1 diabetes and 60% of patients with type 2 diabetes experience diabetic retinopathy [3]. The application of artificial intelligence has led to remarkable performance in collecting eye data and in detecting eye disease [4–6]. Studies have reported that it is feasible to diagnose diabetes using eye data [7, 8].

Traditional Chinese Medicine (TCM) has long been used in China to cure or relieve diabetes. Moreover, various studies have proved the effectiveness of TCM for curing or relieving diabetes [9–12]. Therefore, in this study, physical and physiological features of the patient, namely, the tongue and pulse, are used for diagnosis according to TCM methodology. Specifically, the appearance of the tongue and the pattern of the pulse are combined with fundus photography to diagnose diabetes.

Owing to a technique that uses fundus photography to collect eye data and the effectiveness of TCM in the treatment of diabetes, we combine fundus photography with physical and physiological feature data obtained using monitoring devices. We use the machine learning random forest algorithm for data analysis and for diagnosis.

In this study, we propose a technique for diagnosing both type 1 and type 2 diabetes in a quick, noninvasive way by using equipment that can be easily transported. The proposed method has four main advantages. First, it is easy to use, and the staff using it does not require extensive medical knowledge. Second, it is mobile and can be transported by automobile. Third, it is not likely to cause infection because all the required procedures are noninvasive. Finally, it can serve hundreds of patients in a single day, and results can be obtained in minutes, saving time and resources. These makes it suitable for detecting diabetes in different kinds of situations.

For example, many Chinese people live in remote villages and lack medical resources. They may not be able to be tested for diabetes. Our method provides a feasible way to detect diabetes. This may greatly improve their prognosis, especially if the disease is not yet severe.

Related studies have been conducted on detecting or predicting diabetes; however, most of them use equipment or systems that are inconvenient to transport or unsuitable for providing service to a large number of people [13–16]. However, the technique presented in this study addresses these limitations.

## 2. Materials and Methods

### 2.1. Fundus Screening by Fundus Photography

**Figure 1:**
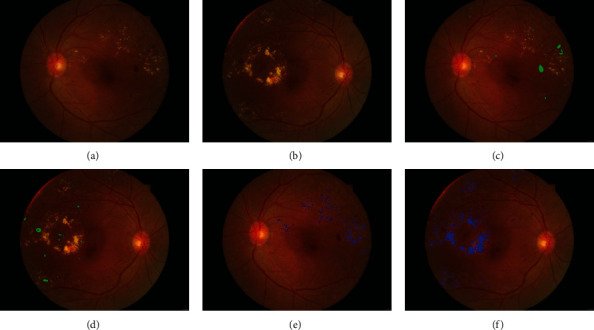
Images depicting hemorrhage and exudation obtained using fundus photography. The blue spotted areas suggest retinal tissue fluid exudation, while the green spotted areas suggest retinal hemorrhage: (a) left eye fundus photography of the subject; (b) right eye fundus photography of the subject; (c) hemorrhage area of the left eye; (d) hemorrhage area of the right eye; (e) exudation area of the left eye; (f) exudation area of the right eye.

With the advancement in computer vision and artificial intelligence, techniques for measuring eye characteristics using fundus photography have been developed. These techniques can be used to detect eye diseases such as diabetic retinopathy or optic nerve hypoplasia [17, 18]. In this study, we detect eye conditions by using fundus photography and the algorithm reported in our previous article [17]. We use the values obtained with the algorithm, which are reported in Table 1.  Figure 1 depicts images of hemorrhage and exudation obtained using fundus photography.

### 2.2. Physical and Physiological Characteristics Relevant to TCM

TCM is a complementary medicine. It has been studied using modern medical methods for decades. As mentioned above, its effectiveness in curing or relieving diabetes has been proven. The characteristics of the human pulse and the appearance of the tongue are two essential factors used for patient examination. These factors were selected in this study to qualify the severity of diabetes.

**Figure 2:**
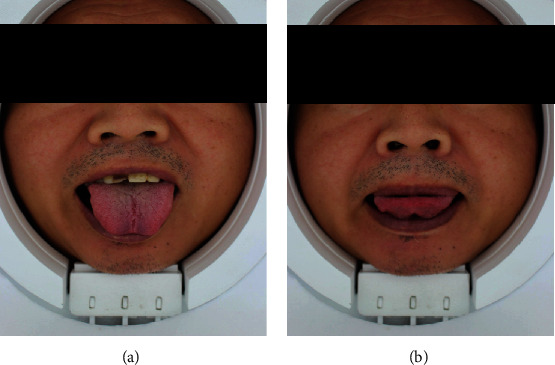
Device for tongue condition detection. In this procedure, the subjects place their face in the detection window. A photo of the face is taken under prescribed illumination conditions. Subsequently, the photo of the tongue is extracted and analyzed by a computer. The computer outputs the detected tongue condition, as listed in Table 2. To ensure that the data was detected correctly, we examined the subject visually under the same conditions of illumination when subjects were not wearing makeup. (a) Front side of the tongue. (b) Back side of the tongue.

**Figure 3:**
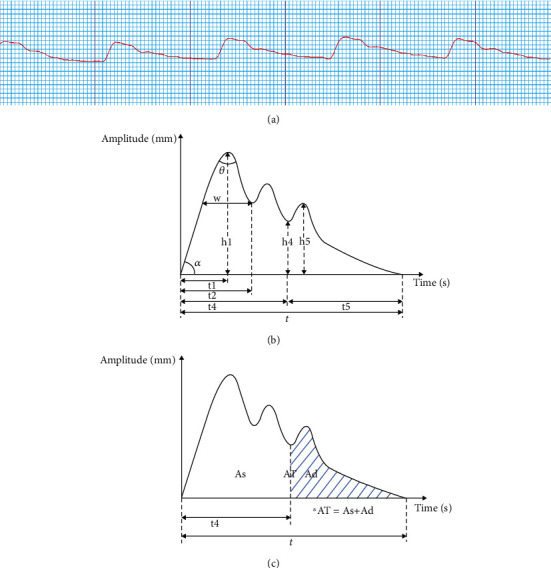
Example of a rough graphic of pulse pattern output and illustration of how the data were measured. Subfigure (a) shows the rough waveform over six cycles. To draw an accurate waveform envelope of the pulse, we measured the amplitude of the subject's pulse over time. Subfigures (b) and (c) show the calculation of the values.

The detection consists of two parts: tongue and pulse conditions. The device used for evaluating the condition of the tongue is shown in Figure 2, while the tongue condition as detected is listed as shown in Table 2. The detected pulse conditions and a rough graphic are shown in Table 3 and Figure 3, respectively.

### 2.3. Dataset

The proposed method was verified experimentally. Macula-centered retinal fundus images were retrospectively obtained from 11 medical institutions in Tianjin, China, by the Ophthalmological Hospital of Tianjin Medical University, of patients who presented for physical examination and retinopathy screening. All images were identified according to the Health Insurance Portability and Accountability Act Safe Harbor prior to transfer to the researchers. This study was approved by the Medical Ethics Committee of Tianjin Medical University Eye Hospital. The subjects authorized the use of the data for this study. No personal information can be recognized or disclosed from the imaged data used in this study. During the enquiry conducted in Tianjin from July 2019 to August 2019, data from 165 subjects were included in a dataset for this study.

### 2.4. Random Forest Algorithm

The random forest algorithm is an “ensemble learning” algorithm, which was first proposed in 2001 [19]. Since then, the method has been widely applied in many domains, and it is considered one of the most powerful machine-learning algorithms [20–23]. The standard procedure for the RF algorithm is as follows [24]:

1. Draw *n*-tree bootstrap samples from the original data
2. For each of the bootstrap samples, grow an unpruned classification or regression tree, with the following modification: at each node, rather than selecting the best split among all predictors, randomly sample *m*_try_ of the predictors and select the best split from among those variables. (Bagging can be considered a special case of random forests obtained when *m*_try_ = *p*, the number of predictors.)
3. Predict new data by aggregating the predictions of the *n*-tree trees (i.e., majority votes for classification or average for regression)

An estimate of the error rate can be obtained based on the training data as follows [24]:

1. At each bootstrap iteration, predict the data not in the bootstrap sample (what Breiman calls “out-of-bag” or OOB data) using the tree grown with the bootstrap sample
2. Aggregate OOB predictions (on average, each data point would be out-of-bag data approximately 36% of the time; thus, aggregate these predictions). Calculate the error rate, and call it the OOB estimate of the error rate

### 2.5. Assessing the Prediction System

In this article, we use a random forest classifier to predict whether a subject has diabetes. A prediction system assessment is needed to validate the prediction of the model. In this regard, an individual instance is classified into one of the following four categories: false positive, true positive, false negative, and true negative. Based on these categories, the total prediction accuracy, precision, recall, and F1 scores for assessment of the prediction system are as follows:

(1) $$\begin{array}{c} \text{Accuracy}=\frac{\text{TP}+\text{TN}}{\text{TP}+\text{TN}+\text{FP}+\text{FN}},\\ \text{Precision}=\frac{\text{TP}}{\text{TP}+\text{FP}},\\ \text{Recall}=\frac{\text{TP}}{\text{TP}+\text{FN}},\\ \text{F}1\text{ score}=\frac{2*\text{precision}*\text{recall}}{\text{precision}+\text{recall}}. \end{array}$$

## 3. Results and Discussion

The results presented in Table 4 show that the proposed method performs properly with an accuracy score of 0.85. Table 4 also lists the result of using fundus photography diagnosis alone. The proposed method scores much higher than fundus photography diagnosis on each aspect.

Comparing the results of the two methods, we assume that the performance improvements are due to the additional health information from the data on the selected characteristics, as well as the robustness of the random forest algorithm. While fundus photography is capable of precision detection, the additional data provides a more general data of a subject's health. However, it has some limitations; for example, the number of subjects used in this study was just 165 so far, which may pose a risk to this study. There is risk of bias inherent in retrospective design. These aspects should be further investigated. We will focus on avoiding these risks.

There are numerous related studies; for instance, references [25–27] focused on the use of artificial intelligence in detecting diabetes; however, these studies mainly focused on software and used hospital data. Other studies [28–30] combined software and hardware, as did our study and were related to diabetes; however, they focused only on diabetic retinopathy, which is a complication of diabetes. Similar studies have attempted to use a convenient and noninvasive method to diagnose diabetes and have achieved an accuracy score higher than 0.85 [31, 32]. Nevertheless, study [31] focused on type 1 diabetes, whereas research [32] focused on type 2 diabetes. Our research mainly focuses on the diagnosis of diabetes, both type 1 and type 2, in a fast and noninvasive way, using equipment that can be transported conveniently. Our method is suitable for serving a large population, especially when the testing locations are not fixed. However, the traditional way to diagnose diabetes, based on plasma glucose criteria, is to measure glycemia in terms of FPG, 2 h PG, and HbA1C (AIC) [33]. Similar to other methods, our method is only a supplemental method and does not replace traditional diabetes diagnosis.

## 4. Conclusions

In this study, we reported a novel method for diagnosing both type 1 and type 2 diabetes in a fast, noninvasive way with equipment that is easy to transport. Our method combined an analysis using fundus photography and particular aspects of the human body, inspired by TCM. The proposed method can be used to diagnose diabetes in many situations, especially in areas where medical resources are lacking, and can serve a large number of people. However, the method is intended for East Asian populations because there is not enough published research showing that TCM also works in populations other than East Asian populations. We will continue this line of research to enrich its practical value, hoping to optimize the method to serve people of other areas.

## Figures and Tables

**Table 1:** Fundus conditions detected using fundus photography. The fundus photography detected the subject's fundus condition. The photographs were then computer analyzed. The fundus characteristics are listed.

Fundus photography report		
	Detection object	Detecting value
Fundus condition	Hemorrhage area (mm^2^)	0.0462
	Hemorrhage lesion number	2
	Maximum hemorrhage area (mm^2^)	0.0308
	Proportion of hemorrhage area	0.0466
	Exudation area (mm^2^)	0.1456
	Exudation lesion number	22
	Maximum exudation area (mm^2^)	0.0182
	Proportion of exudation area	0.1468
	Mean vessel diameter (pixels)	11.527
	Microangioma number	0
	Arterial venous ratio	0.7128
	Cup-disc ratio	0.499
	Average density of leopard spot	0.049
	Atrophic arc and optic disc area ratio	0.547
	Average curvature of vessels	0.0086

Proportion of hemorrhage area: the ratio of the hemorrhage area to the total area taken; proportion of exudation area: the ratio of the exudation area to the total area taken; cup-disc ratio: the ratio of the optic cup area to the optic disc area; average density of leopard spot: the ratio of the total area of leopard spots to the total area taken.

**Table 2:** Example of detected tongue conditions. Three types of physical patterns that can be detected and analyzed are shown: both sides of the tongue and the fur of the tongue. Each characteristic reveals certain aspects of the subject's health condition. For example, a putrid fur on the tongue is a signal of indigestion.

Detected tongue condition	
Feature	Evaluation by TCM
Front side of the tongue: light red, the edge of the tongue is sharp red	Fire in the liver and gallbladder
Fur on the tongue: putrid	Indigestion
Back side of the tongue: normal	QiXue runs smoothly

**Table 3:** Example of detected pulse conditions. The rough waveform of the subject's pulse and the meaning of each detected value are described in Figure 3. The accurate waveform of the pulse was computer analyzed. The waveform data are listed.

Pulse analysis report			
	Detecting potion		Detected value
Pulse shape	Time (s)	*t*	0.84
		t1	0.09
		t2	0.21
		t4	0.34
		t5	0.51
		*w*	0.22
Pulse shape	Amplitude (mm)	h1	8.72
		h4	3.88
		h5	0.11
Pulse shape	Angle	Alpha	74.91
		Theta	77.87
Pulse shape	Area (mm^2^)	AT	69.75
		As	10.72
		Ad	59.02
Pulse shape	Ratio	h1/t1	93.26
		h4/h1	0.44
		h5/h1	0.01
		*w*/*t*	0.26
Pulse shape	Frequency		70
Pulse shape	Analysis	Fluent degree	130.1
		Energy degree	25.66

**Table 4:** Assessing the prediction of the proposed method.

Prediction measures		
	Our proposed diagnosis	Fundus photography alone
Accuracy score	0.85	0.53
Precision score	0.89	0.44
Recall score	0.67	0.39
F1 score	0.76	0.41

## Data Availability

The data cannot be provided because it involves subject privacy.

## References

[1] International Diabetes Federation (2020). *IDF Diabetes Atlas*.

[2] Wu H., Eggleston K. N., Zhong J. (2019). Direct medical cost of diabetes in rural China using electronic insurance claims data and diabetes management data. *Journal of Diabetes Investigation*.

[3] Fong D. S., Aiello L. P., Ferris F. L., Klein R. (2004). Diabetic retinopathy. *Diabetes Care*.

[4] Rahim S. S., Jayne C., Palade V., Shuttleworth J. (2016). Automatic detection of microaneurysms in colour fundus images for diabetic retinopathy screening. *Neural Computing & Applications*.

[5] Rahim S. S., Palade V., Almakky I., Holzinger A., Holzinger A., Kieseberg P., Tjoa A. M., Weippl E. (2019). Detection of diabetic retinopathy and maculopathy in eye fundus images using deep learning and image augmentation. *Machine Learning and Knowledge Extraction, Cd-Make 2019*.

[6] Saha M., Naskar M. K., Chatterji B. N., Chaki R., Cortesi A., Saeed K., Chaki N. (2016). Detection of diabetic retinopathy using the wavelet transform and feedforward neural network. *Advanced Computing and Systems for Security, Vol 1*.

[7] Nangong Z., Hyun K. Method of artificially analyzing iris and retinal images, involves predicting probability of developing diabetes, and predicting type of diabetes based on location and shape of lesion in region of interest.

[8] Ma B. K., Zhao K., Ma S. Y. (2019). Objective analysis of corneal subbasal nerve tortuosity and its changes in patients with dry eye and diabetes. *Chinese Journal of Experimental Ophthalmology*.

[9] Mou X., Zhou D., Zhuang A. W. (2016). Evolution rules of TCM syndrome of patients with type 2 diabetes and diabetic nephropathy. *China Journal of Traditional Chinese Medicine and Pharmacy*.

[10] Wang D., Bai L., Zhao J., Yan W., Li F., Wei J. (2019). Progress in research on newly diagnosed type 2 diabetes with Traditional Chinese Medicine. *Modernization of Traditional Chinese Medicine and Materia Medica--World Science and Technology*.

[11] Xiao E., Luo L. (2018). Alternative therapies for diabetes: a comparison of Western and Traditional Chinese Medicine (TCM) approaches. *Current Diabetes Reviews*.

[12] Oduro P. K., Fang J., Niu L. (2020). Pharmacological management of vascular endothelial dysfunction in diabetes: TCM and western medicine compared based on biomarkers and biochemical parameters. *Pharmacological Research*.

[13] Jayaraman S., Thokala N. K., Purushothaman B. (2020). Device and method to detect diabetes in a person using pulse palpation signal.

[14] Sarno R., Sabilla S. I., Wijaya D. R., Hariyanto (2020). Electronic nose for detecting multilevel diabetes using optimized deep neural network. *Engineering Letters*.

[15] Chi Z., Zhang S., Wang Y., Yang L., Yang Y., Li X. (2016). Research of gestational diabetes mellitus risk evaluation method. *Technology and Health Care*.

[16] Miotto R., Li L., Kidd B. A., Dudley J. T. (2016). Deep patient: an unsupervised representation to predict the future of patients from the electronic health records. *Scientific Reports*.

[17] Xu Y., Wang Y., Liu B. (2019). The diagnostic accuracy of an intelligent and automated fundus disease image assessment system with lesion quantitative function (SmartEye) in diabetic patients. *Bmc Ophthalmology*.

[18] Kruglyakova J., Garcia-Filion P., Nelson M., Borchert M. (2020). Orbital MRI versus fundus photography in the diagnosis of optic nerve hypoplasia and prediction of vision. *British Journal of Ophthalmology*.

[19] Breiman L. (2001). Random forests. *Machine Learning*.

[20] Cheng L., Chen X., De Vos J., Lai X., Witlox F. (2019). Applying a random forest method approach to model travel mode choice behavior. *Travel Behaviour and Society*.

[21] Kim J.-C., Lee S., Jung H.-S., Lee S. (2018). Landslide susceptibility mapping using random forest and boosted tree models in Pyeong-Chang, Korea. *Geocarto International*.

[22] Wager S., Athey S. (2018). Estimation and inference of heterogeneous treatment effects using random forests. *Journal of the American Statistical Association*.

[23] Wright M. N., Ziegler A. (2017). Ranger: a fast implementation of random forests for high dimensional data inC++andR. *Journal of Statistical Software*.

[24] Liaw A., Wiener M. (2002). Classification and regression by randomForest. *R News*.

[25] Liu Y. (2020). Artificial intelligence-based neural network for the diagnosis of diabetes: Model development. *JMIR Medical Informatics*.

[26] Zhu T., Li K., Herrero P., Georgiou P. (2020). Deep learning for diabetes: a systematic review. *IEEE Journal of Biomedical and Health Informatics*.

[27] Ting D. S. W., Pasquale L. R., Peng L. (2019). Artificial intelligence and deep learning in ophthalmology. *British Journal of Ophthalmology*.

[28] Natarajan S., Jain A., Krishnan R., Rogye A., Sivaprasad S. (2019). Diagnostic accuracy of community-based diabetic retinopathy screening with an offline artificial intelligence system on a smartphone. *JAMA Ophthalmology*.

[29] Wang Y.-L., Yang J.-Y., Yang J.-Y., Zhao X.-Y., Chen Y.-X., Yu W.-H. (2020). Progress of artificial intelligence in diabetic retinopathy screening. *Diabetes-Metabolism Research and Reviews*.

[30] Rajalakshmi R., Prathiba V., Arulmalar S., Usha M. (2020). Review of retinal cameras for global coverage of diabetic retinopathy screening. *Eye*.

[31] Czmil A., Czmil S., Mazur D. (2019). A method to detect type 1 diabetes based on physical activity measurements using a mobile device. *Applied Sciences-Basel*.

[32] Hettiarachchi C., Chitraranjan C., Riano D., Wilk S., TenTeije A. (2019). A machine learning approach to predict diabetes using short recorded photoplethysmography and physiological characteristics. *Artificial Intelligence in Medicine, AIME 2019*.

[33] American Diabetes Association (2021). 2. Classification and Diagnosis of Diabetes:Standards of Medical Care in Diabetes—2021. *Diabetes Care*.

